# News and Events of the Week

**Published:** 1910-12-17

**Authors:** 


### News and Events of the Week.

Deputy-Surgeon-General John Martin Hyde, M.D., of Central Hill, Upper Norwood, who died on October 18, aged eighty, left estate of the gross value of £6,836 16s. 9d., with net personalty £6,736.

Sir Alfred Pearce Gould, senior surgeon to the Middlesex Hospital, delivered before the Royal College of Surgeons of England the twenty-ninth annual Bradshaw Lecture last week. He chose "Cancer" for his subject.

Mr. W. E. Hume, M.A., M.B., M.R.C.P., has been appointed to the Lectureship on Materia Medica at the College of Medicine, Newcastle, rendered vacant through the appointment of Dr. Thomas Beattie to the Chair of Therapeutics.

Major Ronald Ross, who was awarded the Nobel Prize for Medicine in 1902, and who is in Stockholm for the celebration of the tenth anniversary of the foundation of the Nobel Institute and for the centenary of the Carolin Institute, the Higher Medical School in Stockholm, has been appointed Honorary Doctor of Medicine. A similar honour has been conferred on M. Emanuel Nobel, nephew of the founder of the Nobel Prizes, who is also attending the two celebrations.

Christmas Presents.—What shall I buy for Christmas presents? is in everyone's mind, or it will be in a few days. For his friends and relatives the medical man has a resource less accessible to the ordinary layman. The Apothecaries' Hall makes excellent scent, and a quarter of a pound of English eau-de-Cologne, lavender water, or Parma violets is usually most acceptable. The present is improved by getting the scent in one of the cut-glass bottles in which the Company puts it up at this time of year.

Mr. James Thomas Macnamara, Medical Officer under the Bermondsey Board of Guardians to the workhouse at Ladywell, has been awarded £250 damages in an action for libel against Messrs. F. Shaw and Co. The passage complained of occurred in the *Southwark and Bermondsey Recorder and South London Gazette* last January. The action was heard in the King's Bench Division of the High Court before Mr. Justice Ridley and a special jury. In the course of his summing up, his lordship stated that there was no institution of to-day which could look after itself better than the Press.

The ninth session of the Australasian Medical Congress will be held in Sydney during September 1911, under the presidency of Mr. F. Antill Pockley, M.B., C.M., Edin., M.B., Syd., M.R.C.S., Eng. The session will commence on Monday, September 18, and end on Saturday, September 23. A social function will be held on the Saturday preceding. The subscription is one guinea. The Railway Departments of Australasia will issue concession tickets to members (with wife or one lady relative accompanying the member) for the return journey at special rates, on presentation of certificates in prescribed form signed by the State Secretaries of Congress. Particulars will be supplied to intending members upon application to State Secretaries. The Inter-State steamship companies have also consented to grant reductions in fares as in previous years.

Dr. Sydney Ringer, M.D., F.R.S., of Lastingham, Yorks, and of Cavendish Place, W., Emeritus Professor of University College, London, who died on October 14, aged seventy-six, left estate valued at £54,521 gross, with net personalty £52,287.

Messrs. Merser and Sons, 268-270 Kennington Road, will shortly publish an English translation of Professor Mosso's *La Paura*, which explains the ætiology, physiology, and pathology of fear. The translation is by Dr. Arthur Harrison, and will be published at 5s. net.

Major James Hamilton Nicholas, R.A.M.C., of St. James's Road, Croydon, who was stabbed mortally by his eldest son, who was suffering from mental derangement, on August 16, left estate now valued for probate at £3,219 10s. 10d. gross, with net personalty nil.

Sir James Sawyer, M.D., of Haseley Hall, Warwick, has presented to the Royal College of Physicians of London a reproduction of the arms of Dr. William Harvey, discoverer of the circulation of the blood. The reproduction has been prepared with considerable labour and research, and a cordial vote of thanks has been passed to Sir James Sawyer for his gift.

An American Society of Medical Sociology has been organised for the purpose of studying radically all questions of a socio-medical nature, and invites co-operation. Among the questions which are under investigation by the members at the present time are: The need of a Federal Department of Health. Tuberculosis as an economic disease. Is there any demonstrable relationship between the strain of our modern life and the increase of insanity? Is cancer on the increase, and if so, what are the probable ætiologic factors? What are the best—*i.e.* the most humane and most effective—methods of dealing with prostitution? The best methods of preventing venereal infection? Is complete sexual abstinence (*a*) likely to impair the general health? (*b*) likely to result in impotence? The results of these investigations will be disseminated by means of meetings, lectures, reports, pamphlets, etc. The Honorary President is Dr. Jacobi, the *doyen* of the profession in America, the President Dr. W. J. Robinson, while Dr. A. C. Jacobson is the Secretary. The offices of the Society are 12 Mount Morris Park, W., New York, from which all particulars and form of membership may be obtained. The subscription is one dollar annually.

Mr. F. R. Anderton, at last week's meeting of the London Education Committee, with Mr. E. A. H. Jay in the chair, submitted a recommendation of the Children's Care (Central) Sub-Committee to renew, subject to the approval of the Board of Education, the agreements entered into with certain London hospitals for the medical treatment of school children. This was carried on a division after Mr. R. Bray's amendment to limit the period of the agreement to six months in each case had been negatived. Mr. Bray's point was that the children were unsympathetically treated at the hospitals and that they had been discharged prematurely after operations and found bleeding in tramcars. Mr. Cyril Jackson said that of the number of children attending the hospitals 85 per cent. continued attendance until they were discharged. Miss K. Wallace thought the scheme of hospital treatment would not work well unless supplemented by school clinics.

The Council of the Royal Institute of Public Health has, Dr. William R. Smith, M.D., writes to the *Times*, recently agreed to take over the work of the Society for the Destruction of Vermin, which will in future be carried on in a special department, under the chairmanship of Sir James Crichton-Browne, M.D., LL.D., F.R.S. In connection with this work the Council desires to undertake researches of a bacteriological and chemical nature, and laboratory facilities will at once be placed at the disposal of the members for any investigation which they may require. The cost of the necessary machinery for the full scheme will be some £10,000, and for this they earnestly appeal. Donations may be paid to the credit of the Institute at the Bank of England (Law Courts Branch), or to the treasurer at the Institute, 37 Russell Square, W.C.

The Charity Commissioners have drafted an order establishing a scheme for the administration of the charity of Muriel Adey, founded by will proved in the Principal Registry on December 24, 1902. The fund, yielding a gross yearly income of about £350, is to be managed by the Principal for the time being of Newnham College, Cambridge, as trustee, or, should she decline to act, by such duly qualified medical woman holding a professional post on the staff of the New Hospital for Women or of the Royal Free Hospital, or of some other hospital in London, as the Charity Commissioners shall from time to time appoint. Subject to payment of expenses, the yearly income is to be applied by the trustee in assisting ladies of small means and of delicate health, provided that such assistance shall be given directly to such ladies and not through institutions, and provided that no person shall be disqualified from receiving such assistance by reason of religious belief or disbelief.

Last week Mr. Troutbeck held an inquest on the body of a labourer of Levens Road, Bow, who had been taken to Poplar Workhouse on November 11 suffering from lung disease and mental trouble. By order of the magistrates he was afterwards removed to Tooting Bec Asylum. Accompanied by an attendant, he was driven on Saturday in a brougham from the Poplar institution, but on the way he was seen to be ill, and brandy was obtained and a doctor called. On arrival at the asylum he was put to bed. Death occurred on Sunday night from heart failure, and the post-mortem examination showed that there was tuberculosis of all the organs. Dr. Donald McRae, medical officer of the Poplar Workhouse, stated that it was not thought there was danger in removing him; but Dr. Turnbull, medical superintendent of the asylum, said that the lungs had been affected for several days, and a long journey in a brougham would tend to make his condition worse. Answering the Coroner, Dr. McRae said the workhouse had an ambulance, and he could have refused his consent to the removal of the man except in that vehicle. Further medical evidence was given to show that there was reason to believe that the removal of the patient in a brougham had hastened his death. The jury returned a verdict that death was due to natural causes, accelerated by the mode of removal, the result of mistaken judgment.

